# Induction with mitomycin C, doxorubicin, cisplatin and maintenance with weekly 5-fluorouracil, leucovorin for treatment of metastatic nasopharyngeal carcinoma: a phase II study

**DOI:** 10.1038/sj.bjc.6690627

**Published:** 1999-08

**Authors:** R L Hong, T S Sheen, J Y Ko, M M Hsu, C C Wang, L L Ting

**Affiliations:** Departments of Oncology, Otolaryngology and Radiation Therapy, National Taiwan University Hospital, National Taiwan University, No. 7 Chung-Shan South Road, Taipei, 10016, Taiwan

**Keywords:** nasopharyngeal carcinoma, metastasis, chemotherapy, mitomycin, cisplatin

## Abstract

The combination of cisplatin and 5-fluorouracil (5-FU) (PF) is the most popular regimen for treating metastatic nasopharyngeal carcinoma (NPC) but it is limited by severe stomatitis and chronic cisplatin-related toxicity. A novel approach including induction with mitomycin C, doxorubicin and cisplatin (MAP) and subsequent maintenance with weekly 5-FU and leucovorin (FL) were designed with an aim to reduce acute and chronic toxicity of PF. Thirty-two patients of NPC with measurable metastatic lesions in the liver or lung were entered into this phase II trial. Mitomycin C 8 mg m^−2^, doxorubicin 40 mg m^−2^ and cisplatin 60 mg m^−2^ were given on day 1 every 3 weeks as initial induction. After either four courses or remission was achieved, patients received weekly dose of 5-FU 450 mg m^−2^ and leucovorin 30 mg m^−2^ for maintenance until disease progression. With 105 courses of MAP given, 5% were accompanied by grade 3 and 0% were accompanied by grade 4 stomatitis. The dose-limiting toxicity of MAP was myelosuppression. Forty per cent of courses had grade 3 and 13% of courses had grade 4 leukopenia. No grade 3 or 4 cisplatin-related toxicity was observed. The overall response rate was 94% (95% confidence interval (CI) 84.9–100%) with a complete response rate (CR) of 6% (95% CI: 0–15.2%) and a good partial response (PR) rate of 28% (95% CI 11.7–44.6%), which was optionally defined as observance of only equivocal lesion identifiable under imaging study. Twenty-seven cases entered weekly FL maintenance phase. The median duration of maintenance with weekly FL was 38 weeks (8–91 weeks). There was no grade 3 or 4 toxicity noted during weekly FL. The median progression-free survival and overall survival were 11.6 ± 0.4 and 18.1 ± 3.6 months respectively. Six patients with a median follow-up of 19.8 months (9.6–41.0 months) were still alive and five of them had disease under control with FL. Good responders (CR and good PR) had better survival than less satisfactory responders (PR and stable disease) (*P* = 0.05). From Cox’s multivariate regression analysis, the only significant prognostic factor for survival was good response to MAP (*P* = 0.042). Liver metastasis was the only significant variable in the best subset regression model that predicted good response to MAP (CR and good PR) (*P* = 0.027). MAP was an effective combination for metastatic NPC with minimal stomatitis and cisplatin-related toxicity but had significant myelosuppression. Weekly FL was a maintenance therapy with minimal side-effects. The response rate and overall survival of MAP-FL were better than series previously reported even when a subset of patients with poor prognosis was selected. MAP-FL's role as neoadjuvant or adjuvant therapy is worthy of further study. © 1999 Cancer Research Campaign


*British Journal of Cancer* (1999) **80**(12), 1962–1967
© 1999 Cancer Research Campaign
Article no. bjoc.1999.0627

### Induction with mitomycin C, doxorubicin, cisplatin and maintenance with weekly 5-fluorouracil, leucovorin for treatment of metastatic nasopharyngeal carcinoma: a phase II study

**RL Hong[1], TS Sheen[2], JY Ko[2], MM Hsu[2], CC Wang[1] and LL Ting[3]**

Departments of [1]Oncology, [2]Otolaryngology and [3]Radiation Therapy, National Taiwan University Hospital, National Taiwan University, No. 7, Chung-Shan South Road, Taipei 10016, Taiwan

**Summary** The combination of cisplatin and 5-fluorouracil (5-FU) (PF) is the most popular regimen for treating metastatic nasopharyngeal carcinoma (NPC) but it is limited by severe stomatitis and chronic cisplatin-related toxicity. A novel approach including induction with mitomycin C, doxorubicin and cisplatin (MAP) and subsequent maintenance with weekly 5-FU and leucovorin (FL) were designed with an aim to reduce acute and chronic toxicity of PF. Thirty-two patients of NPC with measurable metastatic lesions in the liver or lung were entered into this phase II trial. Mitomycin C 8 mg $m^{-2}$, doxorubicin 40 mg $m^{-2}$ and cisplatin 60 mg $m^{-2}$ were given on day 1 every 3 weeks as initial induction. After either four courses or remission was achieved, patients received weekly dose of 5-FU 450 mg $m^{-2}$ and leucovorin 30 mg $m^{-2}$ for maintenance until disease progression. With 105 courses of MAP given, 5% were accompanied by grade 3 and 0% were accompanied by grade 4 stomatitis. The dose-limiting toxicity of MAP was myelosuppression. Forty per cent of courses had grade 3 and 13% of courses had grade 4 leukopenia. No grade 3 or 4 cisplatin-related toxicity was observed. The overall response rate was 94% (95% confidence interval (CI) 84.9–100%) with a complete response rate (CR) of 6% (95% CI: 0–15.2%) and a good partial response (PR) rate of 28% (95% CI 11.7–44.6%), which was optionally defined as observance of only equivocal lesion identifiable under imaging study. Twenty-seven cases entered weekly FL maintenance phase. The median duration of maintenance with weekly FL was 38 weeks (8–91 weeks). There was no grade 3 or 4 toxicity noted during weekly FL. The median progression-free survival and overall survival were $11.6 \pm 0.4$ and $18.1 \pm 3.6$ months respectively. Six patients with a median follow-up of 19.8 months (9.6–41.0 months) were still alive and five of them had disease under control with FL. Good responders (CR and good PR) had better survival than less satisfactory responders (PR and stable disease) ($P = 0.05$). From Cox's multivariate regression analysis, the only significant prognostic factor for survival was good response to MAP ($P = 0.042$). Liver metastasis was the only significant variable in the best subset regression model that predicted good response to MAP (CR and good PR) ($P = 0.027$). MAP was an effective combination for metastatic NPC with minimal stomatitis and cisplatin-related toxicity but had significant myelosuppression. Weekly FL was a maintenance therapy with minimal side-effects. The response rate and overall survival of MAP-FL were better than series previously reported even when a subset of patients with poor prognosis was selected. MAP-FL's role as neoadjuvant or adjuvant therapy is worthy of further study.

**Keywords**: nasopharyngeal carcinoma; metastasis; chemotherapy; mitomycin; cisplatin

Nasopharyngeal carcinoma (NPC) is an endemic disease in Southeast Asia, Taiwan and Southern China. The majority of cases involve Epstein–Barr virus-related non-keratinizing carcinoma, including differentiated and undifferentiated subtypes (Shanmugaratnam et al, 1991). The local control rate of NPC after radiation has been higher than 70% but the distant metastasis rate has been 20–35% at 5 years (Huang et al, 1985; Teo et al, 1989; Lee et al, 1992). When metastases occur, most of these patients succumb within months, especially those with liver and/or lung metastases (Hsu et al, 1983). Combination of cisplatin and 5-fluorouracil (5-FU) (PF) has generally been regarded as the treatment of choice. The response rate of metastatic NPC to PF has been around 60% (Decker et al, 1983; Boussen et al, 1991; Choo et al, 1991; Cvitkovic et al, 1991; Mahjoubi et al, 1992; Gebbia et al, 1993; Su et al, 1993; Chi et al, 1994, 1995; Yeo et al, 1996; Fountzilas et al, 1997).

However, stomatitis, the dose-limiting toxicity, has been very severe and common, possibly because of prolonged 5-FU infusion and prior irradiation (Dreyfuss et al, 1990; Vokes et al, 1990). Chronic toxicities including ototoxicity, nephrotoxicity and neurotoxicity, related to repeated use of high dose cisplatin also become dose-limiting, especially for cases with long-term survival. In addition, patients have to be hospitalized during treatment because of the required 120 h infusion of 5-FU. Outpatient-based therapy with less toxicity and response rates equivalent to or better than PF is urgently needed to improve the quality of life of patients with metastatic NPC.

Mitomycin C has been demonstrated to be synergic with cisplatin in vitro (Teicher et al, 1985; Durand, 1989) and is preferentially activated under hypoxic conditions (Rockwell et al, 1982), which are believed to exist in large tumours. In two studies, mitomycin C increased the response rate significantly when added to

*Received 15 October 1998*
*Revised 4 February 1999*
*Accepted 20 February 1999*

*Correspondence to:* R-L Hong the original protocols for treatment of head and neck squamous cell carcinoma (Kohno et al, 1991; Osoba et al, 1992). Greater than additive cytotoxicity has also been observed for combinations of doxorubicin and cisplatin in multiple experimental system (Drewinko et al, 1976). 5-FU has moderate activity for head and neck squamous cell carcinoma. Its combination with leucovorin, FL is a common treatment for many cancers (Grem et al, 1987). In our institution, a significant portion of NPC patients with metastasis have responded to weekly FL (unpublished data). The administration of weekly FL is convenient and the toxicity is minimal.

Based on the above considerations, we designed a sequential MAP-FL combination protocol for a phase II study. MAP was used for initial induction, aimed especially at hypoxic cells that may have relative drug resistance, as extensive metastasis and huge tumours are very common in NPC (Su et al, 1993). After achieving remission, MAP was followed by weekly administration of FL for maintenance, which took advantage of different action mechanisms and minimal accumulated toxicity. We selected patients with liver and/or lung metastasis, a sub-population with measurable lesions and worst outcome to test feasibility and efficacy.

### PATIENTS AND METHODS

### Patient eligibility

From October 1993 to June 1997, 32 consecutive patients with measurable metastatic liver or lung lesions from NPC were entered onto this phase II study. These patients had histologically confirmed NPC at the primary site. Patients with bone metastasis only were not included because of the difficulty of confirmation and measurability. All patients had a Karnofsky performance scale of 60% or better. Adequate bone marrow function (leucocyte count $\geq$ 3500 $\mu l^{-1}$, platelet count $\geq$ 100 000 $\mu l^{-1}$), liver function (total bilirubin $\leq$ 2.0 mg $dl^{-1}$) and renal function (serum creatinine $\leq$ 2.0 mg $dl^{-1}$) were required. Written informed consents were obtained from study patients.

### Pretreatment work-up

The pretherapeutic work-up included a review of patient's clinical history and a physical examination, head and neck examination and endoscopy, biochemical profile (SMA 12), complete blood cell (CBC) count, erythrocyte sedimentation rate, lactate dehydrogenase, EBV serology (immunoglobulin G [IgG] and IgA), anti-viral capsid antigen (VCA) and early antigen (EA), head and neck computerized tomographic (CT) scan, chest X-ray, bone scintigraphy, abdominal sonography and/or CT scan, and other tests indicated by the clinical picture. This work-up was repeated after two courses of MAP and every 2 months thereafter. Blood chemistries and blood counts were taken before each course to assess renal and haematological toxicity.

### Chemotherapy

The MAP chemotherapy consisted of intravenous administration of mitomycin C 8 mg $m^{-2}$, doxorubicin 40 mg $m^{-2}$ and cisplatin 60 mg $m^{-2}$ on day 1 with hydration and diuresis. Serotonin antagonist and steroids were routinely given for prophylaxis of nausea and vomiting. The cycle was repeated every 3 weeks if haemogram measurements were adequate (leucocyte count $\geq$ 3500 $\mu l^{-1}$ and platelet count $\geq$ 100 000 $\mu l^{-1}$). If leucocyte count was between 3000 and 3500 $\mu l^{-1}$ or platelet count between 75 000 and 100 000 $\mu l^{-1}$ on day 28, the subsequent cycle was modified to a 20% reduction in the dose of mitomycin C and doxorubicin. If leucocyte count was $\leq$ 3000 $\mu l^{-1}$ and platelet count $\leq$ 75 000 $\mu l^{-1}$ on day 28, MAP was withheld until haemogram recovered, then therapy was resumed with a 25% reduction of the dose. MAP was used for induction of remission and a maximum of four courses was given. After MAP, patients were maintained with weekly FL until disease progression. 5-FU 450 mg $m^{-2}$ and leucovorin 30 mg $m^{-2}$ were given weekly. FL was withheld if leucocyte count was $\leq$ 2500 $\mu l^{-1}$ or if there was any evidence of more than grade 2 mucositis or diarrhoea. Cases which had experienced grade 4 non-haematologic toxicity and already achieved a partial response to MAP were allowed to shift to weekly FL before completing four courses of MAP. Cisplatin-containing regimens were used as salvage therapy if disease progression during weekly FL was noted.

Because of the extensive metastasis, radiation was not given to the primary site for those cases with metastasis at initial diagnosis of NPC.

### Toxicity and measurement of response

All toxicities were evaluated at each course of MAP according to the Eastern Cooperative Oncology Group criteria. The evaluation for response was performed with a complete work-up 3 weeks after the second course according to standard World Health Organization (WHO) criteria. Complete response (CR) was defined as disappearance of all detectable malignant disease with no lesion for at least 4 weeks. Partial response (PR) was defined as a more than 50% reduction in the sum of the products of the greatest perpendicular dimensions for all measurable lesions for at least 4 weeks, without new lesions. After chemotherapy, cases with large tumours frequently had only equivocal residual disease in image studies so we optionally defined this condition as good PR. Stable disease (SD) was defined as a decrease of less than 50% or an increase of less than 25% over original measurements of all known lesions.

### Statistical analysis

The survival curves were estimated using the Kaplan–Meier product-limit method (Kaplan and Meier, 1958) Difference between survival curves was tested using the Mantel–Cox log-rank test (Mantel, 1966). The relationship between survival and potential explanatory factors was determined using the Cox proportional hazard technique. The stepwise inclusion procedure was used to determine the statistically significant prognostic factors. The combination of independent variables predicting good response to MAP-FL was evaluated by best subsets regression with software SigmaStat (Jandel Scientific Software).

### RESULTS

### Patient characteristics

A total of 32 patients were included in this study. The characteristics of the patients are summarized in Table 1. Thirty-one cases were WHO type II or III in histology. Three cases presented with metastatic disease upon diagnosis of NPC. Cases with lung or liver

© 1999 Cancer Research Campaign

**Table 1** Patient characteristics

| Characteristics | No |
|---|---|
| Patient number | 32 |
| Age (years) | 44.8 (22–64) |
| Sex (male:female) | 28.4 |
| Karnofsky performance scale | |
| 60 | 2 |
| 70 | 5 |
| 80 | 8 |
| 90 | 17 |
| Histology (WHO type) | |
| I | 1 |
| II | 9 |
| III | 22 |
| Organ involved | |
| Lung | 21 |
| Liver | 18 |
| Bone | 6 |
| Bone marrow | 2 |
| More than one organ | 14 |
| Lesion numbers | |
| < 5 | 11 |
| 5–9 | 6 |
| 10–15 | 15 |
| Largest tumour diameter (cm) | |
| <5 | 20 |
| 5–12 | 12 |

**Table 2** Frequecy of toxicity of MAP combination chemotherapy in 105 courses to 32 patients

| Toxicity (%) | Grade 0 | Grade 1 | Grade 2 | Grade 3 | Grade 4 |
|---|---|---|---|---|---|
| Nausea | 39 | 23 | 27 | 11 | 0 |
| Vomiting | 44 | 19 | 33 | 4 | 0 |
| Stomatitis | 69 | 10 | 16 | 5 | 0 |
| Diarrhoea | 87 | 6 | 5 | 2 | 1 |
| Alopecia | 13 | 9 | 78 | 0 | 0 |
| Leukopenia | 12 | 10 | 24 | 40 | 13 |
| Thrombocytopenia | 59 | 14 | 12 | 11 | 3 |
| Hepatotoxicity | 84 | 10 | 5 | 0 | 1 |
| Nephrotoxicity | 87 | 11 | 1 | 0 | 1[a] |
| Infection | 84 | 6 | 5 | 4 | 2 |

[a] Haemolytic uraemic syndrome.

**Table 3** Response rate to MAP in metastatic NPC

| | Number | % | 95% confidence interval |
|---|---|---|---|
| RR | 30 | 94 | 84.9–100 |
| CR | 2 | 6 | 0.0–15.2 |
| Good PR | 9 | 28 | 11.7–44.6 |
| PR | 19 | 59 | 41.4–77.4 |
| SD | 2 | 6 | 0.0–15.2 |
| PD | 0 | 0 | 0.0–0.0 |

RR: response rate; CR: complete remission; PR: partial remission; SD: stable disease; PD: progressive disease; good PR: only equivocal lesion(s) remained in image study.

**Table 4** Best model predicting response to MAP from best subsets regression

| Variable[a] | Coefficient | Standard error | *t* Statistic | *P*-value | Variance inflation factor |
|---|---|---|---|---|---|
| Constant | –0.630 | 0.736 | –0.856 | 0.399 | 0.000 |
| Liver | 0.365 | 0.156 | 2.340 | 0.027 | 1.127 |
| Alb | 0.304 | 0.181 | 1.677 | 0.105 | 1.193 |
| LDH | –0.00018 | 0.000105 | –1.716 | 0.098 | 1.490 |
| TTR | –0.000173 | 0.000123 | –1.413 | 0.169 | 1.415 |

*R squared = 0.365, adjusted R square = 0.271. [a]Variables used including age, sex, performance status, metastatic sites, tumour diameter, haemoglobin, albumin, LDH, time to tumour relapse after radiotherapy (TTR).

metastasis were 21 and 18 respectively. Fourteen cases had more than one organ involvement. Twenty-one cases had more than five metastatic lesions and 12 cases had largest tumour diameters of more than 5 cm. Twenty-six cases showed at least one of these two features of advanced disease. The mean lactate dehydrogenase (LDH) level was 1030 U $l^{-1}$ (270–3350) (normal 230–460), and 20 cases had abnormally high LDH levels. Three patients had prior exposure to cisplatin or mitoxantrone as part of induction or adjuvant chemotherapy.

### Toxicity of MAP

One hundred and five cycles of MAP were administered to 32 patients. The mean dosages given were 96.0 ± 7.5, 96.0 ± 5.4 and 97.3 ± 5.6 (mean ± s.d.) of planned doses for mitomycin C, doxorubicin and cisplatin respectively. Nausea and vomiting was generally not severe with the prophylaxis of a serotonin antagonist (Table 2). Stomatitis was common, but only 5% of the courses were accompanied by grade 3 and 0% accompanied by grade 4 stomatitis, even though the majority of cases had previously received radiation of 7000 cGy to the nasopharynx and neck. The major toxicity was myelosuppression, and 40% and 13% of courses had grades 3 and 4 leukopenia respectively. Two severe infection episodes occurred during MAP chemotherapy and one of them died of sepsis. Another case had haemolytic uraemic syndrome related to the use of mitomycin C, which subsided after appropriate treatment. No grade 3 or 4 cisplatin-related toxicity was observed.

### Response to MAP

The response results are summarized in Table 3. Of the 32 patients studied, two (6%) CR, nine (28%) good PR, and 19 (59%) PR were observed with an overall response rate of 94% (95% CI

 *© 1999 Cancer Research Campaign*

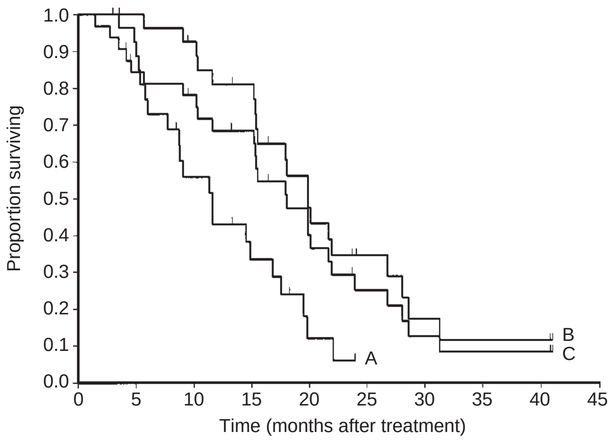

**Figure 1** Kaplan–Meier plots of progression-free survival (curve A), disease-specific survival (curve B) and overall survival (curve C) of 32 patients with metastatic NPC who received MAP induction and weekly FL for maintenance

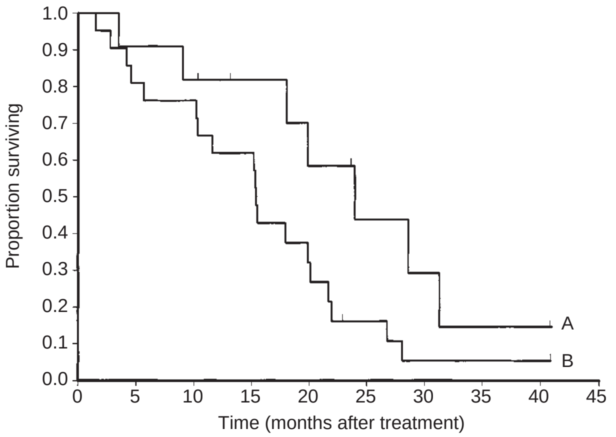

**Figure 2** Kaplan–Meier plots of survival grouped by response to MAP as good response (CR plus good PR; curve A) versus less satisfactory response (PR plus SD; curve B). The difference between these two curves was tested by Mantel–Cox log-rank test ($P = 0.05$)

84.9–100%). Two cases were categorized as stable disease and no case had disease progression during MAP.

By best subsets regression, liver metastasis was the only significant variable in the best subset model that predicted good response to MAP (CR and good PR; $P = 0.027$, Table 4). All metastatic lesions in liver responded well to MAP, but the duration of response maintained by weekly FL varied greatly, which is reflected on the wide variation of progression-free periods (Figure 1). Serum LDH level, although generally accepted to reflect the tumour burden, did not correlate with response to MAP. Time to tumour relapse after radiotherapy, theoretically related to the rate of tumour growth, also did not correlate with response.

### Maintenance treatment with FL and survival

Excluding two cases with SD, one case with haemolytic uraemic syndrome, one case died of sepsis and another case shifted to usage of traditional herb drug, 27 cases entered the weekly FL maintenance phase. The median duration of maintenance with weekly FL was 38 weeks (8–91 weeks). There was no grade 3 or 4 toxicity noted during weekly FL. Three patients were still in remission with treatment of weekly FL for 13, 14 and 21 months respectively. Three deaths occurred while the diseases were still in remission, but were not chemotherapy-related. Two patients died of choking and aspiration pneumonia at 3 and 15 months of weekly FL therapy, very likely due to poor upper aerodigestive tract function, which is a common chronic side-effect of radiotherapy for NPC. Another patient, with disease at good remission for 2 years, died of vertebra-basilar artery ischaemia, which was thought to be a complication of radiation.

The progression-free survival, overall survival and disease-specific survival are shown in Figure 1 and the medians were $11.6 \pm 0.4$, $18.1 \pm 3.6$ and $19.9 \pm 0.3$ months respectively. Six cases with a median follow-up of 19.8 months (9.6–41.0 months) were still alive. In addition to the three cases maintained in remission with FL, two cases once had disease progression due to interruption of FL treatment, still had disease under control with weekly FL and had already survived 25 and 41 months respectively.

None of the six cases whose disease progressed within 4 months of weekly FL responded to salvage cisplatin-based chemotherapy. In contrast, among the ten cases with FL maintenance for at least 4 months, eight of them responded to cisplatin-based therapy at disease progression ($P = 0.066$ by Fisher's exact test).

Good responders (CR and good PR) to MAP had better survival than less satisfactory responders (PR and stable disease) ($P = 0.05$, Figure 2). Using Cox's proportional hazard model for multivariate analysis, including age, sex, performance status, metastatic sites, tumour diameter, haemoglobin, albumin, LDH, response to MAP and courses of MAP as independent variables, the only statistically significant factor for survival was good response to MAP ($P = 0.042$). Metastatic sites or tumour load which was reflected by LDH and tumour diameter did not correlate with survival.

### DISCUSSION

The previously reported response rates of metastatic NPC to cisplatin-based chemotherapy ranged from 40% to 83.8% and the complete remission rate from 4% to 24.3%. The averaged response rate was 59.6% (95% CI 54–65%) and the CR rate was 17.6% (95% CI 13.3–21.9%) (Table 5) (Decker et al, 1983; Boussen et al, 1991; Choo et al, 1991; Cvitkovic et al, 1991; Mahjoubi et al, 1992; Gebbia et al, 1993; Su et al, 1993; Chi et al, 1994, 1995; Yeo et al, 1996; Fountzilas et al, 1997). Cases with complete remission had relatively long survival, especially cases with bone metastasis (Cvitkovic et al, 1991; Su et al, 1993), but the prognosis of non-responders were grave (Hsu et al, 1983). The median survival, available only from a limited number of reports, ranged from 11.4 months to 16 months. How to further increase response rate and how to decrease toxicity of treatment are important issues.

The results of the present study were quite encouraging. Compared with previously reported series, MAP-FL had a significantly higher response rate and the longest median survival reported to date (Table 5), even though we selected a subset of patients with large tumour burden and unfavourable metastatic sites. All cases with liver metastasis achieved good response with

© 1999 Cancer Research Campaign

**Table 5** Summary of cases of metastatic NPC treated with cisplatin-based chemotherapy[a]

| Authors | Date | Drug used | Case no. | RR (%) | 95% CI | CR (%) | 95% CI | Median survival |
|---|---|---|---|---|---|---|---|---|
| Foutzilas et al | 1997 | Carboplatin/paclitaxel | 14 | 57.1 | 27.5–86.8 | 14.3 | 0.0–35.3 | 15 |
| Yeo et al | 1996 | Carboplatin/5 FU | 42 | 38.1 | 22.8–53.4 | 16.7 | 4.9–28.5 | 12.1 |
| Chi et al | 1995 | Cisplatin/5FU/LV | 17 | 70.6 | 46.5–94.7 | 23.5 | 1.0–46.0 | 16 |
| Chi et al | 1994 | Cisplatin/5FU/LV | 15 | 80.0 | 57.1–100.0 | 13.3 | 0–32.8 | 14 |
| Su et al | 1993 | Cisplatin/5FU/bleomycin | 25 | 40.0 | 19.4–60.6 | 4.0 | 0–12.3 | n.a. |
| Gebbia et al | 1993 | Cisplatin-based | 15 | 77.0 | 48.0–98.1 | 33.0 | 6–60 | 11.4 |
| Mahjoubi et al | 1992 | Cisplatin/epirubicin/bleomycin | 44 | 50.0 | 34.6–65.4 | 20.0 | 7.6–32.4 | n.a. |
| Choo et al | 1991 | Cisplatin-based | 30 | 70.0 | 51.0–85.0 | 23.3 | 7.2–39.4 | n.a. |
| Boussen et al | 1991 | Cisplatin/5FU/bleomycin | 37 | 83.8 | 71.3–96.3 | 24.3 | 9.8–38.8 | n.a. |
| Cvitokovic et al | 1991 | Cisplatin/epirubicin/mitomycin C/5-FU | 46 | 61.0 | 46.2–75.3 | 8.7 | 0.2–17.2 | n.a. |
| Decker et al | 1983 | Cisplatin based | 17 | 52.9 | 26.5–79.5 | 17.7 | 0–37.9 | n.a. |
| All | | | 302 | 59.6 | 54.0–65.2 | 17.6 | 13.3–21.9 | |
| Hong et al | 1999 | MAP-FL | 32 | 94.0 | 85.0–100 | 34.4[b] | 17.0–52 | 18.1 |

[a]Only cases with metastatic disease were included in the study[b] Including CR and good PR (only equivocal lesion(s) in image study). RR: response rate; CR: complete remission; NA: not available; 95% CI: 95% confidence interval.

MAP and liver metastasis was the only significant variable to predict good response to MAP. From a previous larger series, the mean survival time for liver and lung metastases were 5.4 ± 0.5 months (mean ± standard error), and 11.8 ± 1.8 months respectively (Hsu et al, 1983). In the present study, the mean survival for cases with liver and lung metastasis was 19.3 ± 7.7 and 19.6 ± 4.7 months respectively. Response to chemotherapy was the only significant prognostic factor and there was no difference in survival with respect to metatastic sites and tumour burdens. A more effective therapy may change the prognostic factors defined from series treated with less active protocols.

In the present study, the toxicity of MAP was acceptable. Grades 3 and 4 stomatitis were lower compared to those commonly observed more than 80% seen in PF for head and neck cancers (Dreyfuss et al, 1990; Vokes et al, 1990; Chi et al, 1994). Furthermore, with a lower dosage of cisplatin and fewer courses of cisplatin-based chemotherapy, nephrotoxicity, ototoxicity and neurotoxicity were minimal. However, the myelotoxicity of MAP was significantly higher. Use of mitomycin C accounted for the accumulated myelotoxicity and haemolytic uraemic syndrome. Reducing the courses of MAP and shifting to weekly FL earlier may help to ameliorate myelotoxicity. The number of MAP courses did not significantly influence the outcome or survival, but MAP was very effective in quickly reducing tumour load, especially for cases with particularly heavy tumour loads. The median duration of remission maintained by weekly FL was 38 weeks. Not including three disease-free cases died of radiation-related toxicity, five of the 27 cases entered FL had disease maintained in SD or in remission with a median of 14.5 months. There were no grade III or IV toxicities noted during FL therapy. Considering the low toxicity and reasonable activity, weekly FL was a good maintenance therapy.

Several new chemotherapeutic drugs are now available, but the data concerning their activity in NPC is extremely lacking. Use of single agent paclitaxel and combination paclitaxel and cisplatin showed an unsatisfactory response rate (Table 5) (Au et al, 1996; Fountzilas et al, 1997). Cisplatin, which is inexpensive and effective, is still the cornerstone for treatment of metastatic NPC. New drugs or new combination developments in treating NPC should be aimed at reducing side-effects, especially accumulated toxicity.

The approach in the present study of using MAP for induction and weekly FL for maintenance had a satisfactory high response rate and longer remission periods than those reported from previous studies. The synergism between mitomycin C, doxorubicin and cisplatin might have contributed to the high response rate noted in our study. Maintenance with weekly FL may generally reduce the toxicity of cisplatin-based combinational chemotherapy. This protocol is effective, inexpensive and feasible for outpatient therapy. We are now conducting a clinical trial to incorporate this treatment into the initial treatment of advanced NPC, hoping to improve overall survival.

### REFERENCES

Au E, Ang PT and Chua EJ (1996) Paclitaxel in metastatic nasopharyngeal cancer. *Proc Am Soc Clin Oncol* **15**: 322–322

Boussen H, Cvitkovic E, Wendling JL, Azli N, Bachouchi M, Mahjoubi R, Kalifa C, Wibault P, Schwaab G and Armand JP (1991) Chemotherapy of metastatic and/or recurrent undifferentiated nasopharyngeal carcinoma with cisplatin, bleomycin, and fluorouracil. *J Clin Oncol* **9**: 1675–1681

Chi KH, Chan WK, Cooper DL, Yen SH, Lin CZ and Chen KY (1994) A phase II study of outpatient chemotherapy with cisplatin, 5-fluorouracil, and leucovorin in nasopharyngeal carcinoma. *Cancer* **73**: 247–252

Chi KH, Chan WK, Shu CH, Law CK, Chen SY, Yen SH and Chen KY (1995) Elimination of dose limiting toxicities of cisplatin, 5-fluorouracil, and leucovorin using a weekly 24-hour infusion schedule for the treatment of patients with nasopharyngeal carcinoma. *Cancer* **76**: 2186–2192

Choo R and Tannock I (1991) Chemotherapy for recurrent or metastatic carcinoma of the nasopharynx. A review of the Princess Margaret Hospital experience. *Cancer* **68**: 2120–2124

Cvitkovic E, Bachouchi M and Armand JP (1991) Nasopharyngeal carcinoma. Biology, natural history, and therapeutic implications. *Hematol Oncol Clin North Am* **5**: 821–838

Day RS (1986) Treatment sequencing, asymmetry, and uncertainty: protocol strategies for combination chemotherapy. *Cancer Res* **46**: 3876–3885

Decker DA, Drelichman A, al-Sarraf M, Crissman J and Reed ML (1983) Chemotherapy for nasopharyngeal carcinoma. A ten-year experience. *Cancer* **52**: 602–605

Drewinko B, Green C and Loo TL (1976) Combination chemotherapy in vitro with cisdichlorodiammineplatinum(II). *Cancer Treat Rep* **60**: 1619–1625

Dreyfuss AI, Clark JR, Wright JE, Norris CMJ, Busse PM, Lucarini, JW, Fallon BG, Casey D, Andersen JW and Klein R (1990) Continuous infusion high-dose leucovorin with 5-fluorouracil and cisplatin for untreated stage IV carcinoma of the head and neck. *Ann Int Med* **112**: 167–172

Durand RE (1989) Synergism of cisplatin and mitomycin C in sensitive and resistant cell subpopulations of a tumor model. *Int J Cancer* **44**: 911–917

 *© 1999 Cancer Research Campaign*

Fountzilas G, Skarlos D, Athanassiades A, Kalogera-Fountzila A, Samantas E, Bacoyiannis C, Nicolaou A, Dombros N, Briasoulis E, Dinopoulou M, Stathopoulos G, Pavlidis N, Kosmidis P and Danilidis J (1997) Paclitaxel by three-hour infusion and carboplatin in advanced carcinoma of nasopharynx and other sites of the head and neck. A phase II study conducted by the Hellenic Cooperative Oncology Group. *Ann Oncol* **8**: 451–455

Gebbia V, Zerillo G, Restivo G, Speciale R, Cupido G, Lo BP, Ingria F, Gallina S, Spatafora G and Testa A (1993) Chemotherapeutic treatment of recurrent and/or metastatic nasopharyngeal carcinoma: a retrospective analysis of 40 cases. *Br J Cancer* **68**: 191–194

Grem JL, Hoth DF, Hamilton JM, King SA and Leyland-Jones B (1987) Overview of current status and future direction of clinical trials with 5-fluorouracil in combination with folinic acid. *Cancer Treat Rep* **71**: 1249–1264

Hsu MM and Tu SM (1983) Nasopharyngeal carcinoma in Taiwan. Clinical manifestations and results of therapy. *Cancer* **52**: 362–368

Huang SC, Lui LT and Lynn TC (1985) Nasopharyngeal cancer: study III. A review of 1206 patients treated with combined modalities. *Int J radiat Oncol Biol Phys* **11**: 1789–1793

Kaplan EL and Meier P (1958) Nonparametric estimation from incomplete observations. *J Am Stat Soc C* **53**: 457–481

Kohno N, Inuyama Y, Sakurai S and Ohnuma T (1991) BOMM regimen for the treatment of advanced head and neck carcinoma. *Cancer Invest* **9**: 485–489

Lee AW, Poon YF, Foo W, Law SC, Cheung FK, Chan DK, Tung SY, Thaw and Ho JH (1992) Retrospective analysis of 5037 patients with nasopharyngeal carcinoma treated during 1976–1985: overall survival and patterns of failure. *Int J Radiat Oncol Biol Phys* **23**: 261–270

Mahjoubi R, Azli N and Bachouchi M (1992) Metastatic undifferentiated carcinoma of nasopharyngeal type treated with bleomycin (B), epirubicin (E) and cisplatin (C): final report. *Proc Am Soc Clin Oncol*, **11**, 240–240.

Mantel N (1966) Evaluation of survival data and two new rank order statistics arising in its consideration. *Cancer Chemother Rep* **50**: 163–170

Osoba D, Band PR, Connors JM, Goldie JH, Knowling MA, Fetherstonhaugh and EM (1992) Treatment of recurrent and metastatic head and neck cancer with cisplatin/etoposide/bleomycin. *Semin Oncol* **19**: 25–29

Rockwell S, Kennedy KA and Sartorelli AC (1982) Mitomycin-C as a prototype bioreductive alkylating agent: in vitro studies of metabolism and cytotoxicity. *Int J Radiat Oncol Biol Phys* **8**: 753–755

Shanmugaratnam K and Sobin L (1991) *Histological Typing of Tumors of the Upper Respiratory Tract and Ear. WHO International Histological Classification of Tumors*. Springer-Verlag: Berlin

Su WC, Chen TY, Kao RH and Tsao CJ (1993) Chemotherapy with cisplatin and continuous infusion of 5-fluorouracil and bleomycin for recurrent and metastatic nasopharyngeal carcinoma in Taiwan. *Oncology* **50**: 205–208

Teicher BA, Gunner LJ and Roach JA (1985) Chemopotentiation of mitomycin C cytotoxicity in vitro by platinum complexes. *Br J Cancer* **52**: 833–839

Teo P, Tsao SY, Shiu W, Leung WT, Tsang V, Yu P and Lui C (1989) A clinical study of 407 cases of nasopharyngeal carcinoma in Hong Kong. *Int J Radiat Oncol Biol Phys* **17**: 515–530

Vokes EE, Schilsky RL, Weichselbaum RR, Kozloff MF and Panje WR (1990) Induction chemotherapy with cisplatin, fluorouracil, and high-dose leucovorin for locally advanced head and neck cancer: a clinical and pharmacologic analysis. *J Clin Oncol* **8**: 241–247

Yeo W, Leung TW, Leung SF, Teo PM, Chan AT, Lee WY and Johnson PJ (1996) Phase II study of the combination of carboplatin and 5-fluorouracil in metastatic nasopharyngeal carcinoma. *Cancer Chemother Pharmacol* **38**: 466–470

*© 1999 Cancer Research Campaign*
